# Supplementary material for: Exploring white matter dynamics and morphology through interactive numerical phantoms: the White Matter Generator
Source: Front Neuroinform. 2024 Jul 31;18:1354708. doi: 10.3389/fninf.2024.1354708 (PMC11322502; doi:10.3389/fninf.2024.1354708)
Supplement: Supplementary file 1 [file Image_1.pdf]

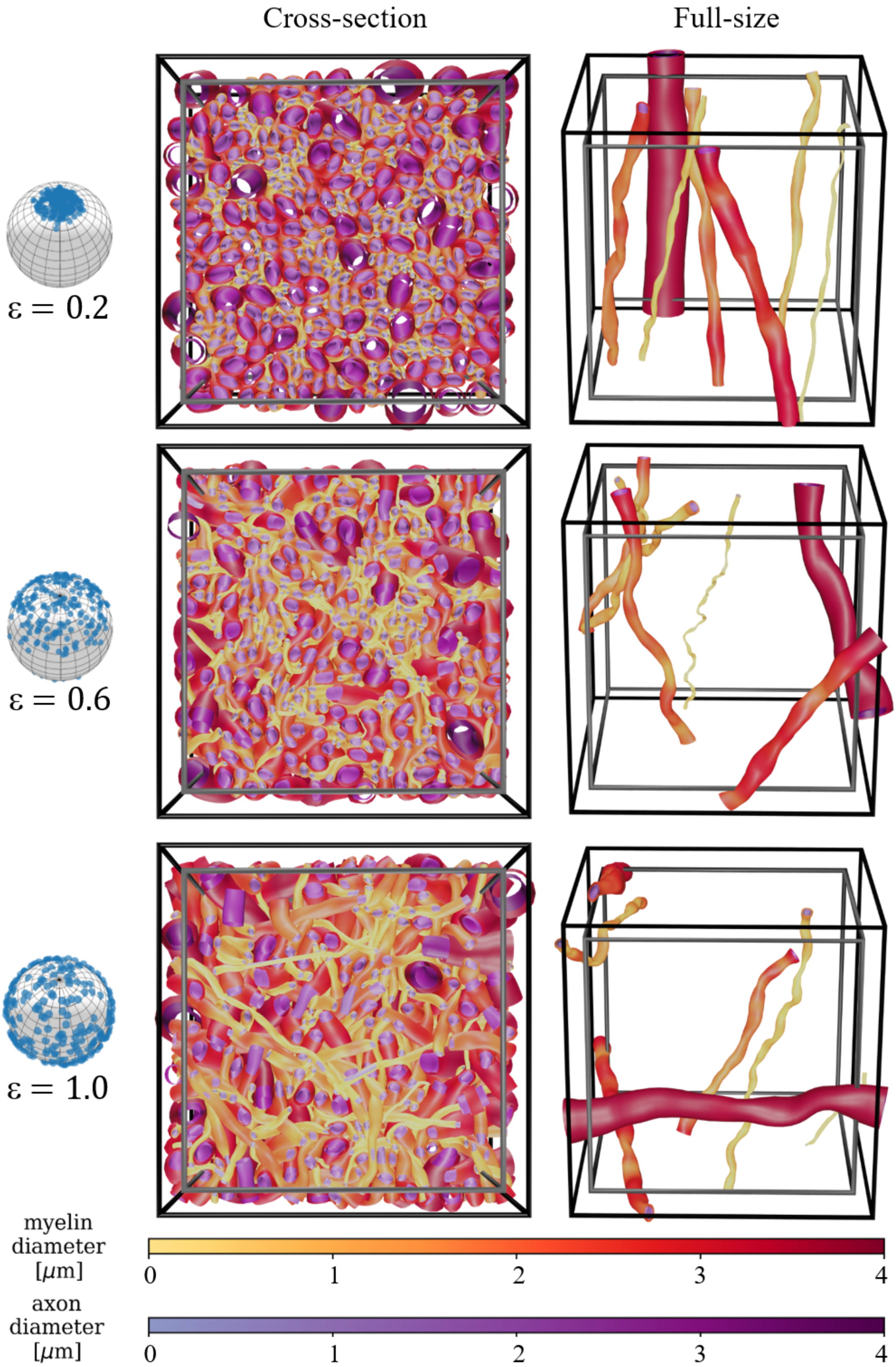

Figure S1: Demonstration of the global fibre dispersion parameter ϵ. The phantoms shown here are the same as shown in Fig. 4 with CVF=0.00. Each phantom is generated from the same axon diameter distribution but with different degrees of ϵ. The unit spheres on the left show the global dispersion associated with each ϵ. For the cross-sectional view (**left column**), all axons are cut at 1/3 of the voxel's height. For the full-size view (**right column**), seven fibre IDs were randomly selected for a more explicit visualisation of their individual morphologies.
